# The Impact of HIV Genetic Polymorphisms and Subtype Differences on the Occurrence of Resistance to Antiretroviral Drugs

**DOI:** 10.1155/2012/256982

**Published:** 2012-06-26

**Authors:** Mark A. Wainberg, Bluma G. Brenner

**Affiliations:** Jewish General Hospital AIDS Centre, McGill University, 3755 Cote-Ste-Catherine Road, Montreal, QC, Canada H3T 1E2

## Abstract

The vast majority of reports on drug resistance deal with subtype B infections in developed countries, and this is largely due to historical delays in access to antiretroviral therapy (ART) on a worldwide basis. This notwithstanding the concept that naturally occurring polymorphisms among different non-B subtypes can affect HIV-1 susceptibility to antiretroviral drugs (ARVs) is supported by both enzymatic and virological data. These findings suggest that such polymorphisms can affect both the magnitude of resistance conferred by some major mutations as well as the propensity to acquire certain resistance mutations, even though such differences are sometimes difficult to demonstrate in phenotypic assays. It is mandatory that tools are optimized to assure accurate measurements of drug susceptibility in non-B subtypes and to recognize that each subtype may have a distinct resistance profile and that differences in resistance pathways may also impact on cross-resistance and the choice of regimens to be used in second-line therapy. Although responsiveness to first-line therapy should not theoretically be affected by considerations of viral subtype and drug resistance, well-designed long-term longitudinal studies involving patients infected by viruses of different subtypes should be carried out.

## 1. Introduction

Nonsubtype B infections are responsible for most HIV cases worldwide [1]. HIV-1 group M has been classified into subtypes, circulating and unique recombinant forms (CRF and URF, resp.), due to its significant natural genetic variation; this includes subtypes A–D, F–H, and J–K and many CRFs and URFs. Although subtype B is the most prevalent in the Western World (Western Europe, the Americas, Japan, and Australia), non-B subtypes predominate in the rest of the world: that is, subtype C in sub-Saharan Africa, India, and Brazil, CRF01_AE in South East Asia, CRF02_AG in West Africa, and subtype A in Eastern Europe and Northern Asia [1–3]. The proportion of non-B subtypes in North and South America and Western Europe is increasing [4–7]. Combination antiretroviral therapy (ART) is now used in many areas of the world, and HIV resistance to antiretroviral drugs (ARVs) has widely emerged. Thus, non-B subtypes will presumably become even more common in western countries.

Reduced sensitivity to ARVs in non-B subtypes has been less well studied than in subtype B, mainly because of the predominance of subtype B in those countries in which ARVs first became available, coupled with the availability of genotypic and phenotypic antiretroviral drug resistance testing in such countries [8]. This notwithstanding there is a potential for genetic differences among subtypes to yield differential patterns of resistance-conferring mutations in response to ARVs and this possibility is supported by the fact that HIV-1 naturally varies in genetic content by as much as 35% among subtypes. Indeed, variation is higher in some areas of the genome (40% in the env gene) and lower in others (8–10% in the *pol*, *gag,* and *IN* genes) [8]. Since differences in codon sequences at positions associated with drug resistance mutations might predispose viral isolates from different subtypes to encode different amino acid substitutions, it is possible that HIV-1 genetic diversity may influence the types of resistance mutations that might eventually emerge upon drug exposure as well as the rate of emergence of such mutations and phenotypic resistance [8, 9]. Such diversity may also affect the degree of cross-resistance to ARVs of the same class, with the potential to impact on virologic failure, clinical outcomes, and preservation of immunological responsiveness [8].

For example, studies of single dose nevirapine (sdNVP) for prevention of mother-to-child transmission (PMTCT) showed a disparity in overall resistance among subtypes, with frequencies of 69, 36, 19, and 21% against NVP in women with subtypes C, D, A, and CRF02_AG infections, respectively. Often, this result occurred prior to treatment and despite the absence of resistance mutations [10–13]. Very sensitive PCR detection procedures, which reveal resistance due to minority species, have revealed a higher incidence of NVP resistance (K103N, Y181C) in 70–87% of individuals with subtype C compared with 42% of individuals with subtype A [14–16].

Evaluations of virological and biochemical data also suggest that natural amino acid background can affect the magnitude of resistance conferred by many mutations responsible for antiretroviral drug resistance [17], as is best illustrated by HIV-2 and group O viruses that show high-level innate resistance to nonnucleoside reverse transcriptase inhibitors (NNRTIs) through the presence of natural polymorphisms that can confer drug resistance (Table 1) [18, 19]. However, many studies on antiretroviral drug resistance in non-B subtypes exposed to chronic suppressive therapy have yielded less definitive results with respect to the importance of natural HIV-1 diversity as a factor leading to differences in types of drug resistance mutations and the propensity to develop drug resistance in the first place [8, 17].

 Although genotypic ARV resistance testing is of proven benefit in deciding on best choice of ARVs for individual treatment and serves as a repository of information on HIV resistance mutations, several factors underscore the difficulties in defining intersubtype differences. For example, genotyping can classify the major viral subtypes, but significant proportions (~15%) of infections remain unassigned or differentially assigned using different subtyping algorithms [8, 20, 21]. Certainly, HIV resistance databases make efforts to incorporate newer subtype data into pools of data, but the availability of HIV genotypes from areas of the world with non-B subtype predominance is still comparatively low [22]. The factors responsible include lesser availability of ARV therapy, the high cost of drug resistance testing, and limited opportunities for research in resource-limited areas. In some cases, resistance tests may often be performed only on participants enrolled in study cohorts or trials but not in general practice.

## 2. Resistance to Nucleoside Reverse Transcriptase Inhibitors (NRTIs)

As an example of disparity, subtype C patients in Botswana treated with ZDV/ddI developed an atypical thymidine analogue mutation (TAM) resistance pathway (67N/70R/215Y) compared to subtype B (the TAM 1 and TAM 2 pathways) [23]. This distinction was not observed in patients with subtype C in Malawi, India, or South Africa [24–27]. Results from Botswana also reported a high incidence of K65R (30%) in subtype C patients who received d4T/ddI plus NVP or efavirenz (EFV) [28]. A much larger study from Malawi detected K65R or K70E in 23% of patients failing first-line therapy with d4T/3TC/NVP [26], while K65R was detected in 7% and 15% of patients in South Africa failing first- or second-line regimens, respectively, whose nucleoside backbones included d4T/3TC or ddI/ZDV [29, 30]. A study from Israel also reported a high frequency of K65R in subtype C viruses from Ethiopian immigrants [31], and a report from India showed that K65R was present in about 10–12% of patients who had received d4T/3TC/NVP in first-line therapy [32]. Such differences in K65R and thymidine analogue mutations (TAMs) might be attributed to treatment regimen and disease stage [24–27].

Access to viral load testing lead in India was also associated with early detection of NRTI-treatment failure, leading to use of new, second-line regimens and preventing acquisition of TAMs and K65R [24]. Additional studies support regional differences among subtype C subepidemics from Ethiopia, Brazil, and sub-Saharan Africa, that impact on NRTI resistance rates as a result of different NRTI-based regimens [8, 33, 34].

**Figure 1:**
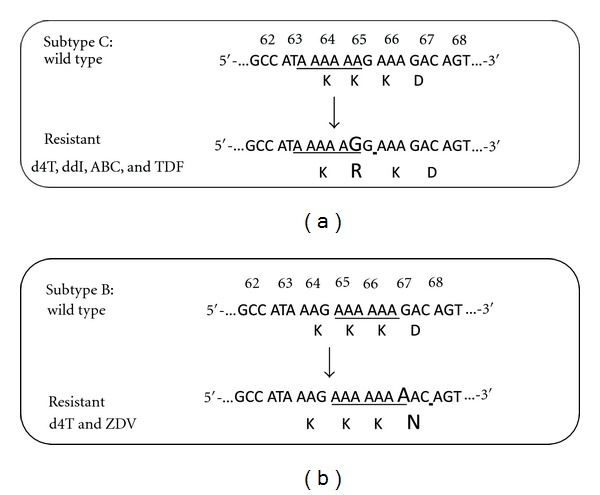
Subtype-specific poly-A nucleotide motifs lead to template pausing under pressure with thymidine analogues that favor K65R selection in subtype C and D67N selection in subtype B. Depiction of the template-based propensity of subtype C versus B viruses to develop the K65R mutation that is associated with broad cross-resistance among multiple members of the NRTI family of drugs. The codons located at positions 63, 64, and 65 in subtype C RT seem to be critically involved in the preferential development of K65R in subtype C. d4T: stavudine, ddI: didanosine, ABC: abacavir, TDF: tenofovir. It should be noted that the use of stavudine in particular has been shown to yeild K65R in subtype C infections with high frequency. Regimens that are based on the use of TDF and ABC, among other drugs, can help mitigate the development of the K65R mutation.

Higher rates of the K65R mutation in subtype C [26, 28, 29] suggest that these viruses may have a particular predisposition toward acquiring this mutation [35]. A subtype C RNA template mechanism has been proposed to explain this phenomenon that involves higher rates of K65R mutagenesis in subtype C viruses than in other subtypes (Figure 1) [36, 37], and this mechanism seems to be template dependent and is independent of the source of the reverse transcriptase (RT) employed [36]. Subtype C viruses apparently have an intrinsic difficulty in synthesizing stretches of adenine homopolymeric runs that leads to template pausing at codon 65, facilitating the acquisition of K65R under selective drug pressure [37, 38], whereas the subtype B template favors pausing at codon 67 that may facilitate the generation of D67N and TAMs rather than K65R [37–39]. In addition, the introduction of codons from positions 64 and 65 in the RT of subtype C into a subtype B backbone was sufficient to lead to selection of K65R by multiple NRTIs [37–39].Figure 1 provides a pictorial representation of the preferential development of K65R in subtype C viruses.

Ultrasensitive pyrosequencing has also been used to detect the spread of K65R as transmitted and/or minority species in treatment-naïve populations [40, 41]. Patients harboring subtype C infections showed a higher frequency of K65R than subtype B variants (1.04% versus 0.25%) by this method but these differences were not duplicated using limiting dilution clonal sequencing approaches [40]. While these findings are consistent with PCR-induced pausing, leading to low-level spontaneous generation of K65R in subtype C, they do not negate the higher rates of development of K65R in subtype C populations failing regimens containing d4T, ddI, or tenofovir (TDV) [32]. The occurrence of K65R in subtype C and CRF01_AE is also associated with the Y181C NVP mutation within the viral backbone [30, 42].

 Subtype C selected the K65R mutation in drug resistance selection studies faster than subtype B under TFV pressure [35]. However, K65R may be less frequent in subtype A than other subtypes [43]. And a higher propensity to acquire TAMs was reported in patients carrying CRF_06 (AGK recombinants) as compared to patients carrying CRF02_AG from Burkina Faso [44].

The differential selection of K65R pathways in subtype C seems related to template differences, ddI and d4T-containing regimens, as well as to the presence of Y181C. Further genotypic studies will be required to ascertain subtype differences in acquisition of resistance to NRTIs.

## 3. Resistance to Nonnucleoside Reverse Transcriptase Inhibitors (NNRTIs)

Selection studies in culture have shown that a V106M mutation commonly develops in subtype C viruses following drug pressure with NVP or EFV, whereas a V106A mutation is more commonly selected in subtype B. This difference is due to a nucleotide polymorphism at codon 106 in RT [45, 46], and the clinical importance of V106M in non-B subtypes has been confirmed in multiple studies showing that V106M is frequently seen in non-B subtypes (C and CRF01_AE) after therapy with NVP or EFV [23, 25, 27, 47–50].

The G190A substitution was also relatively more frequent among subtype C infected patients failing NNRTI-based therapy in Israel and India, and G190A/S was seen in the Israeli study as a natural polymorphism in subtype C from Ethiopian immigrants [25, 49]. The frequencies of these mutations among treated patients in both studies were higher than in subtype B and C drug-naïve individuals.

Although the overall prevalence of V106M in subtype C is higher than in subtype B (12% versus, 0%) in individuals failing NNRTI-based regimens, K103N (29% versus 40%) and Y181C (12% versus 23%) remain important pathways for both subtype C and B, respectively (hivdb.stanford.edu). Only minor differences in HIV resistance pathways seem to occur among subtypes A, B, and C with the second generation NNRTI etravirine (ETR) [50]. 

## 4. PR Mutations

The results of work with protease inhibitors PIs indicate that the D30N mutation was not observed in CRF02_AG and CRF02_AE isolates in patients failing nelfinavir (NFV) therapy but rather that the N88S mutation emerged after NFV use in CRF01_AE and after indinavir [51] use in subtype B [52, 53]. Although another study reported an absence of the D30N mutation in CRF01_AE, no information on the specific type of PIs received by the patients was available [54]. A lower frequency of D30N was seen in subtype C isolates from Ethiopian immigrants to Israel after NFV usage than in subtype C viruses from Botswana [55, 56], suggesting that subtype C viruses from Ethiopia (the origin of the samples identified in Israel) and southern Africa might behave in different fashion. M89I/V mutations were observed in F, G, and C subtypes but not in other subtypes [26], and the V82I natural polymorphism in subtype G led to the emergence of I82M/T/S in treatment failure [57]. The L90M mutation is rare in subtype F but common in subtype B from Brazil [58], and a recent paper suggests that polymorphisms at position 36 in PR may be important in determining the emergence of specific patterns of resistance mutations among viruses of different subtypes [59].

To gain an understanding of the underlying mechanisms leading to the overall higher preponderance of D30N in subtype B relative to other subtypes, molecular dynamic simulations were performed. D30N appeared to selectively confer resistance to NFV in subtype B by increasing the flexibility of the protease (PR) flap region and destabilizing the PR inhibitor complex [60]. In subtype C, D30N required the accessory N83T mutation to confer resistance and rescue fitness [61].

Two comprehensive surveys reported differences in natural protease polymorphisms among non-B subtypes [62, 63] and positions less frequently mutated in non-B subtypes than in subtype B after exposure to ARVs. Residues of importance in subtype A in PR were at positions 10, 20, and 63, whereas, in subtype C, they were at residues 20, 53, 63, 74, and 82. Other differences were at residues 13 and 20 in subtype D, residues 10, 14, 20, and 77 in subtype F, residues 20, 67, 73, 82, and 88 in subtype G, residues 20, 63, 82, and 89 in CRF01_AE, and residue 20 in CRF02_AG [63].

 Higher rates of accumulation of NRTI and PI resistance mutations and equal rates of emergence of NNRTI mutations were also found in subtype B compared to C [64]. A study from southern Brazil also showed a lower frequency of primary resistance to PIs in subtype C compared to subtype B, suggesting that PI mutations may be less well tolerated at the structural level in subtype C [65].

 However, HIV-1 subtype diversity has not limited the overall benefit of ART (Table 1). This notwithstanding there are subtype differences in the type and preference of pathways of resistance with some mutations emerging almost exclusively in some non-B subtypes, for example, the protease mutation 82 M in subtype G versus 82A/F/S in the others, 88D in subtype B versus 88S in subtypes C and CRF02_AG [66]. Furthermore, HIV-2 has major mutations in regard to NRTIs, NNRTIs, and PIs, which contribute to innate NNRTI resistance and rapid development of multiclass drug resistance (Table 1) [67, 68]. The V106M RT mutation in subtypes C and A versus V106A in subtype B is observed with resistance against NVP and EFV. Polymorphisms at RT residue 98, common in subtype G, are associated with NNRTI resistance in subtype B and may lower the resistance barrier and duration of efficacy of some NNRTIs [69]. The frequency of some resistance mutations shared by B and non-B subtypes can vary after failure of first-line therapeutic regimens, as in the case of the K65R mutation. Differences in type and frequency of resistance mutations should not be underestimated. However, the TAM pathway 67N/70R/215Y found in subtype C in Botswana will probably be adequately detected by most resistance algorithms, since it does not involve new mutations.

 A lower risk for accumulation of major (primary) resistance mutations in subtype C than B has been reported [64]. The major mutations that emerged in both subtypes were the same. Since both subtypes B and C patients had similar profiles of virological failure after use of the same ART regimens, this rules out ancillary factors responsible for these differences. Minor mutations in subtype B PR may appear as frequent natural polymorphisms in several non-B subtypes (e.g., M36I, L89M) [58, 59]. The fact that the L89M polymorphism can lead to the M89I mutation that confers resistance to PIs suggests that there might be a lower accumulation of major mutations in C subtypes, if natural polymorphisms act similarly in subtype C as they do when present as secondary resistance mutations in subtype B.

The majority of non-B HIV-1 subtype isolates possess wild-type susceptibilities similar to those of subtype B wild-type isolates. Compared to B subtypes, diminished susceptibilities among wild-type isolates have been found for CRF02_AG recombinant viruses in three different studies in regard to ATV and NFV [63, 69, 70]. No study has yet assigned statistical significance of drug susceptibility levels due to polymorphisms and small sample size. One analysis performed molecular modeling and suggested that distortions in the K26 pocket of A/G proteases appear to be responsible for a lower binding energy of NFV and hence lower susceptibility of A/G viruses to this drug [70]. A/G isolates with lower susceptibilities to certain PIs (NFV and atazanavir (ATV)) have also been found. One study has detected an important proportion of WT isolates with lower susceptibilities to ATV [71]. In most cases, phenotypes have been determined by commercial or in-house assays that were developed primarily to measure B-subtype drug susceptibilities based on the laboratory adapted strains NL4-3 or HXB2, through use of a modified clone of a laboratory strain that lacks both the terminal part of Gag and most of Pol. It should be recognized that most commercial assays do not monitor polymorphisms, and indeed sequences that lie within particular regions, such as the substrates of PR within gag or the RNaseH and connection domains within pol, can influence drug resistance in both B and non-B subtypes but may not be easily recognized. Although some work has been carried out in this field, it is clear that other studies are required [72–75].

There are few data on the potential for cross-resistance to PIs among non-B subtypes in regard to NFV, although there is a tendency to select for the L90M pathway instead of D30N in subtype C. Competition fitness assays support the notion that subtype C viruses bearing D30N are impaired in replicative fitness, a finding that may explain the above results [61].

Thermodynamic studies performed on target-inhibitor interactions in PR have specifically described a lower affinity of non-B subtype proteases for PIs and amplification of primary resistance mutations on the basis of polymorphisms that are present in background.

In addition to the foregoing, interesting results on polymorphisms that confer hypersusceptibility to some PIs have been recently reported [76]. Some of these polymorphisms can potentially delay acquisition of drug resistance and may therefore enhance the long-term effectiveness of relevant drugs.

## 5. Integrase Inhibitors and Drug Resistance

New data are emerging that subtype differences are also present in regard to integrase strand transfer inhibitors (INSTIs) despite the fact that HIV-1 subtype B and C wild-type integrase (IN) enzymes are similarly susceptible to clinically approved INSTIs [77–81]. This notwithstanding there are now data to indicate that the presence of resistance mutations may differentially affect susceptibility to specific INSTIs in viruses of different subtypes [77]. Moreover, such data have been obtained both in tissue culture using recombinant viruses of different subtypes that contain specific IN mutations as well as in biochemical integrase strand transfer and integrase 3′synthetase assays, in which specific drug resistance mutations have been introduced into recombinant purified integrase enzymes derived from either subtype B or subtype C viruses [77].

Of particular interest may be that a novel next-generation INSTI termed MK-2078 with a higher genetic barrier for selection of resistance than either raltegravir (RAL) or elvitegravir (EGV) was able to differentially select for a novel G118R substitution in IN in subtype C compared with subtype B viruses [82]. This mutation conferred only slight resistance to MK-2048 but gave rise to 25-fold resistance against RAL when it was present together with a polymorphic substitution at position L74M in CRF02-AG cloned patient isolates [83]. It is also well known that INSTI Q148RHK resistance mutations that affect susceptibility to a novel INSTI, dolutegravir (DTG) in HIV-1 subtype B may not affect susceptibility of subtype C viruses or HIV-2 viruses and IN enzymes to the latter compound [84].

Finally, tissue culture selection with DTG has identified a novel R263K resistance mutation in subtype B but not subtype C viruses [85]. In contrast, the same series of selections with DTG in subtype C viruses yielded the same G118R mutation that had previously been obtained with MK-2048, also in subtype C. This raises the possibility that G118R may have the potential to be an important resistance mutation for next-generation INSTIs in subtype C viruses but that this role may be played by R263K in the context of subtype B viruses. Of course, definitive information on this topic may have to await the widespread clinical use of DTG and the characterization of mutations within IN that may arise in the event of rising viral loads and treatment failure.

## 6. Clinical Practice

HIV resistance in non-B subtypes has rarely been reported on the basis of single drugs or NRTI backbones but, rather, mutations have been reported for specific drug classes. Cross-resistance can be estimated only for some NRTIs and NNRTIs but not for most PIs that are the only drugs eligible as part of second-line regimens in most regions of the world. The potential for cross-resistance to NFV in viruses of CRF01_AE and CRF02_AG origin could be higher than has been observed in subtype B, due to the preferential selection of the N88S and L90M substitutions, although such data are not yet available for most PIs in the context of non-subtype B viruses. NRTI backbones may also vary in the mutation profile they select for according to drug combinations that are used. Newer compounds (e.g., TFV and ATV/r) are now preferred both in resource-rich countries and non-B subtype prevalent areas. Although HIV resistance databases continue to enter HIV genotype data from nonB subtype variants, few data sets are available to date (stanford HIV resistance database, Agence Nationale pour la Recherche sur le SIDA-France (ANRS), etc.) for drugs that have become part of first-line therapy in developed countries, for example, TDF, ATV, darunavir, ETR, and RAL. In this context as well, it is relevant that some studies have attempted to address the clinical impact of HIV diversity on treatment response as well as the limitations of such approaches [86, 87].

## 7. Future Considerations

The preferential emergence of some mutations and changes in the frequency of these mutations in select non-B subtypes needs greater attention and research on the role of polymorphisms in nonsubtype B viruses that increase in frequency after drug exposure and that may contribute to drug resistance (e.g., A98G/S in RT and M36I and K20I in PR) [88] should be priorized, particularly in parts of Africa in which treatment failure has been reported in as many as 40% of patients after two years [89] and in India where resistance rates of 80% to two drug classes have been reported after failure of first-line regimens that employed various NRTI/NNRTI combinations [90]. To date, no study has tested the degree of resistance or cross-resistance that certain mutational combinations (67N/70R/215Y) may confer in tissue culture. Newer studies should assess pre- and posttreatment genotypes in order to determine associations of certain polymorphisms with drug resistance, including variations of polymorphisms in variants of the same subtype that are located in different geographical regions. This would improve the appropriateness of use of certain drugs over others in the context of second- or third-line therapeutic regimens.

The different studies conducted in populations affected by nonsubtype B viruses are too heterogeneous to permit pooling of data [8]. Such studies have addressed different research questions and used nonequivalent NRTI backbones (e.g., ZVD/ddI and ZDV/3TC) and have also grouped mutations by drug class without providing information on the nature of the regimen at virologic failure. Resistance has also been reported in different ways (e.g., different algorithms or resistance lists), making it difficult to relate resistance mutations to a specific drug or combination of drugs. More longitudinal studies on response to first-line ARV combinations are needed to better recognize intersubtype differences. Pre- and posttherapy genotype resistance testing is also desirable.

## 8. Conclusions

Virological and biochemical data provide compelling evidence on the differential effect of genetic background on both the type and degree of HIV-1 antiretroviral drug resistance. Genetic background can affect the degree of protein binding caused by primary mutations and restore the function of PR to a differential degree in different subtypes based on background polymorphisms, although this effect was not discernible in the absence of typical major resistance mutations but rather when particular backgrounds of combinations of major resistance mutations and background polymorphisms were represented. Clearly, some background polymorphisms can act as secondary resistance substitutions.

Phenotypic assays have failed to find differences of large magnitude in the susceptibilities of HIV B versus non-B subtypes, consistent with what has been learned at a molecular level. Unfortunately, only few datasets exist on relative susceptibility levels among subtypes carrying specific major resistance mutations, and more information is required, particularly because many polymorphisms in non-B viruses are considered to be secondary resistance mutations since they can emerge after drug exposure in subtype B viruses. The effect of such polymorphisms within different genetic backgrounds cannot always be extrapolated to non-B subtypes and might sometimes contribute to higher levels of resistance depending on genetic backbone. They could also have either a neutral effect or hypersensitize HIV to ARVs, and I93L is an example of a secondary resistance mutation in subtype B that in subtype C causes hypersusceptibility to PIs [61].

Novel NNRTI resistance mutations in subtype C were not recognized in subtype B. In tissue culture, subtype C can acquire a V106M mutation under NNRTI drug pressure compared to V106A in subtype B. V106M can confer broad cross-resistance to an extent that supersedes that conferred by V106A.

The acquisition of resistance could have important implications in regard to durability of therapy. In culture, the emergence of the K65R mutation is quicker in subtype C than in B [30, 35], and several biochemical mechanisms have been proposed to explain this observation, based on subtype C templates [36–38, 91]. K65R has been seen in approximately 70% of patients failing ddI-containing nucleoside backbones in Botswana [28] but does not appear to emerge frequently in subtype C patients who have received either TDF or TDF/FTC as part of triple therapy [30], a possible reflection of the use of well-tolerated effective drugs that have long mutually reinforcing intracellular half-lives that act in combination to suppress viral replication and prevent the emergence of resistance mutations. Higher numbers of patients and longer followup will be required to determine if there is a consistent impact of subtype C in the emergence of K65R in the clinic.

Multiple *in vitro* and clinical studies have confirmed that PR and Gag can act as a functional unit and coevolve when HIV is subject to drug pressure. Both genes can clearly mutate under PI pressure, and Gag mutations can act as compensatory substitutions that may increase levels of viral replication capacity and resistance. The recombinant phenotyping systems used for clinical samples do not now adequately monitor Gag. While differences among Gag may vary between only −2 to 2.5-fold between subtypes, different subtypes might develop compensatory Gag mutations at different rates, establishing a need to take Gag into account in determining a phenotype. One study reported that a recombinant construct included Gag of clinical origin but did not test the same subtypes as were reported in other work [57].

Although various mutations can impact on drug sensitivity to differential extent, such information cannot yet be generated with regard to non-B subtypes due to a paucity of paired phenotypic and genotypic data. Three studies analyzed genotypes and phenotypes of non-B subtypes in clinical trials: one on use of single dose NVP for prevention of mother-to-child transmission and two on double and triple NRTI combinations that are no longer used [8].

Cross-resistance acquires importance in settings with limited access to antiretroviral therapy, and few *in vitro* comparative data are available for PIs in non-B subtypes. However, such data may be crucial to understanding cross-resistance to specific drugs [58, 59], since some PIs may be the only potentially accessible option for drug sequencing in salvage therapy in many resource-limited settings. The fact that resistance to PIs commonly requires that large numbers of resistance mutations be present may yield a situation in which the individual contribution of any single mutation to drug resistance, with some exceptions, will be limited, a definite advantage of using drugs with a high genetic barrier toward the development of drug resistance. Thus, differences among subtypes with regard to development of drug resistance are more likely to be important for NRTIs and NNRTIs than for PIs. Clearly, large numbers of paired samples need to be systematically collected from naïve and treated patients infected with subtypes C, AE, AG, A, and G, in order for genotypic and phenotypic analysis to be conducted for both established drug classes as well as for newer classes of drugs such as inhibitors of integrase.

Finally, this paper has focused on classes of HIV drugs for which significant datasets are available in regard to subtypes and differential drug resistance. Limitations of both space and available datasets have precluded us from discussing the topic of entry inhibitors. However, most available data suggest that the only two approved entry inhibitors, that is, the fusion inhibitor, enfuvirtide, and the CCR5 entry antagonist, maraviroc, are both active against HIV isolates of multiple subtypes.

## Figures and Tables

**Table 1:** Examples of polymorphisms and mutations in reverse transcriptase (RT), protease (PR), and integrase (IN) of different subtypes that may impact on emergent resistance to nucleoside and nonnucleoside reverse transcriptase inhibitors (NRTIs and NNRTIs), protease inhibitors (PIs), and integrase strand transfer inhibitors (INSTIs).

Drug class	Type/group/ subtype	Polymorphism or mutation associated with drug resistance	Drug(s) affected	Mutation(s) and their consequences	Reference
Reverse transcriptase					

NRTI	C	64-65-66 KKK motif	ddI, d4T, TDF	K65R	[30]
NRTI	HIV-2	T69N, V75I, V118I, L210N, T215S, K219N	NRTIs	TAMs/K65R	[66]
NNRTI	C	V106V	EFV, NVP	V106M	[45]
NNRTI	G	A98S	NNRTIs		[66]
NNRTI	HIV-2	Y181I,Y188L, G190A K101A, V106I, V179I	All NNRTIs	Cross- NNRTI resistance	[68]
NNRTI	O	Y181C, A98S, K103R, V179E	All NNRTIs	Cross- NNRTI resistance	[18]

Protease					

PI	Non-B	M36I	PIs		[59]
PI	G, AE	K20I	PIs		[63]
PI	G	V82I	PIs	I82M/T/S	[63]
PI	A, C, F, G, AE, AG	L89M	PIs	L89I	[71]
PI	HIV-2	L10I/V, K 20V, V32I, M36I, M46I, I47V, L63E/K, A71V, G73A, V77T, V82I/L,	PIs	APV and other PIs	[68]

Integrase					

INSTIs	B	R263	MK-2048, DTG	R263K	[85]
	C	G118	MK-2048, DTG	G118R	[82]

ddI: didanosine; d4T: stavudine; TFV, tenofovir: EFV, efavirenz: NVP, nevirapine: DTG, dolutegravir.
